# Differences in Chemical Constituents between *Dalbergia oliveri* Heartwood and Sapwood and Their Effect on Wood Color

**DOI:** 10.3390/molecules27227978

**Published:** 2022-11-17

**Authors:** Liuming Wei, Ruoke Ma, Yunlin Fu

**Affiliations:** College of Forestry, Guangxi University, Nanning 530004, China

**Keywords:** *Dalbergia oliveri*, heartwood, wood color, pigment composition

## Abstract

The purpose of this study was to characterize and quantify the chemical constituents of heartwood and sapwood of *Dalbergia oliveri* extract in order to investigate the chemical components that determine the formation of heartwood’s color. In this work, the types of pigments in heartwood and sapwood extract were analyzed using UV-Visible (UV) Spectrophotometer, and the main pigment components of heartwood and sapwood extract were identified and quantified using ultra-high performance liquid chromatography-mass spectrometry (UHPLC-MS). The results showed that the difference in content of the main components between heartwood and sapwood of *Dalbergia oliveri* was slight, and the lignin structure between heartwood and sapwood is basically identical; flavonoid pigments were found to be the primary chromophoric components of heartwood and sapwood extract. However, a total of 21 flavonoids were identified in heartwood and sapwood, of which the unique substances to heartwood were vitexin, isorhamnetin, and pelargonidin, and the content of isoliquiritigenin, formononetin, and biochanin A were 253, 37, and 583 times higher in the heartwood than in the sapwood, respectively, which could be the main pigment components affecting the significant color difference between heartwood and sapwood of *Dalbergia oliveri*. These results will provide a foundation for revealing the underlying mechanism of color difference between heartwood and sapwood and provide a theoretical basis for wood coloring.

## 1. Introduction

*Dalbergia oliveri* Gamble ex Prain (*D. oliveri*) belongs to the genera of *Dalbergia*, a family of Fabaceae, and is commercially known as Burmese tulipwood, which is mainly distributed in Myanmar, Thailand, Vietnam, Laos, etc. It was included in the International Union for Conservation of Nature (IUCN) Red List of Threatened Species in 1998 [1]. Sapwood and heartwood are yellow and light red or reddish brown, respectively. Compared to sapwood, heartwood has texture, strong natural durability, fragrance, and potential medicinal value [2,3,4]. Therefore, it has always been used as a material for high-class furniture, which has been loved by people. As market demand increased, many of the original sources of rosewoods have been indiscriminately harvested and drastically reduced. As a result, the price of rosewood increased rapidly. As an example, *Dalbergia bariensis* timber originating from Vietnam is 3478.3–3913.0 USD/m^3^, while imported *Dalbergia bariensis* timber is 1787.0 USD/m^3^ [5]. To cater for changing market needs, fast-growing and abundant plantation timber (eucalyptus, poplar) has become an alternative in China because of its short rotation period and low price, but the drawbacks are poor decay resistance effect and monotone color of the wood [6,7]. Color affects the visual aesthetics and decorative qualities of wood and wood products, which is an important indicator to evaluate the ornamental and commercial value. Natural dyes are used in textile dyeing and food coloration due to their fewer toxins, UV resistance, and antibacterial properties [8,9]. It is worth noting that natural dyes can be an effective solution to the problem of monotonous wood color, but their few sources, high cost, and poor water fastness have restricted the development of natural dyes in wood dyeing, which is still in the experimental research stage [10,11]. Zhu et al. [12,13,14] reported that natural dyes extracted from residues of *Dalbergia cochinchinensis* using ethanol exhibited desirable color appearance, water fastness, and permeability, as well as having some anti-UV properties and mold-inhibition properties in wood staining, and natural dyes from other plants have been repeatedly discussed in the literature [11,15]. A large number of residues (shavings, trimmings) are left over in the production and processing of rosewood. It is often used as fuel, resulting in a large number of wasted resources. Thus, natural dyes extracted from rosewood residues are applied to fast-growing woods. It not only improves the utilization rate of rare rosewood resources, but also increases the added value of fast-growing wood.

The difference in heartwood and sapwood color and natural durability is mainly due to the changes in chemical composition and contents during the formation of heartwood [8,16]. Previous studies have shown that wood color is closely related to the chemical composition, which mainly included flavonoids, anthraquinones, and phenolic compounds [16,17,18]. Therefore, it is important to explore the chemical composition that determines the formation of wood color for the preparation technology of wood dyeing. Including affluent chemical components, the extractives of *D. oliveri* have the potential value of providing natural dyes. Liu et al. [19] probed *D. oliveri* extractives by using gas chromatography-mass spectrometry (GC-MS) and finally detected a total of 32 compound components, with relative contents of more than 1% and high alcohol and ketone levels. However, the relationship between the chemical composition of *D. oliveri* extractives and wood color has not been clarified.

Based on the above issues, this study aimed to investigate the correlation between the chemical composition of *D. oliveri* extracts and wood color and to reveal the cause for the formation of color in *D. oliveri* wood. For this purpose, we processed the waste of *D. oliveri* wood as the research material, and ultraviolet-visible (UV) spectroscopy was used to analyze the main types of chromophores. They were identified and quantified by ultra-high performance liquid chromatography-mass spectrometry (UHPLC-MS).

## 2. Results and Discussion

### 2.1. Main Chemical Components Analysis of D. oliveri Sapwood and Heartwood

#### 2.1.1. Main Chemical Component Contents and PH Value of Sapwood and Heartwood

The main component contents and PH value of heartwood and sapwood of *D. oliveri* are shown in Table 1. It can be seen that the content of holocellulose in heartwood of *D. oliveri* was 8.54% lower than that in sapwood. The content of α-cellulose in heartwood was 3.18% higher than that in sapwood, and the Klason lignin content in heartwood was slightly higher than that in sapwood, by 0.34%. The acid-insoluble lignin was slightly higher in heartwood. The acid-soluble lignin was just lower than sapwood. The results for holocellulose and α-cellulose in sapwood and heartwood are consistent with those reported in the literature for *Eucalyptus globulus* (21.1–24.1% and 43.4–46.1%), but *Dalbergia oliver* has a higher content than that of *Eucalyptus globulus* [20]. The results were similar to those of other studies, including the Klason lignin content in sapwood and heartwood of *Quercus faginea* (28.1% and 28.6%, respectively), *Quercus Laurina* (25.1% and 25.5%, respectively), and *Quercus crassifolia* (24.9% and 25.2%, respectively) reported in the literature [21,22]. Lignin macromolecules contain chromophoric groups such as benzene rings, carbonyl vinyl groups, and auxochrome groups such as hydroxyl, carboxyl, and ether bonds, which can give wood its color. Therefore, the difference in the functional group structure between heartwood and sapwood requires further characterization and analysis to explore its effect on color.

#### 2.1.2. Characterization of Klason Structure

**Figure 1:**
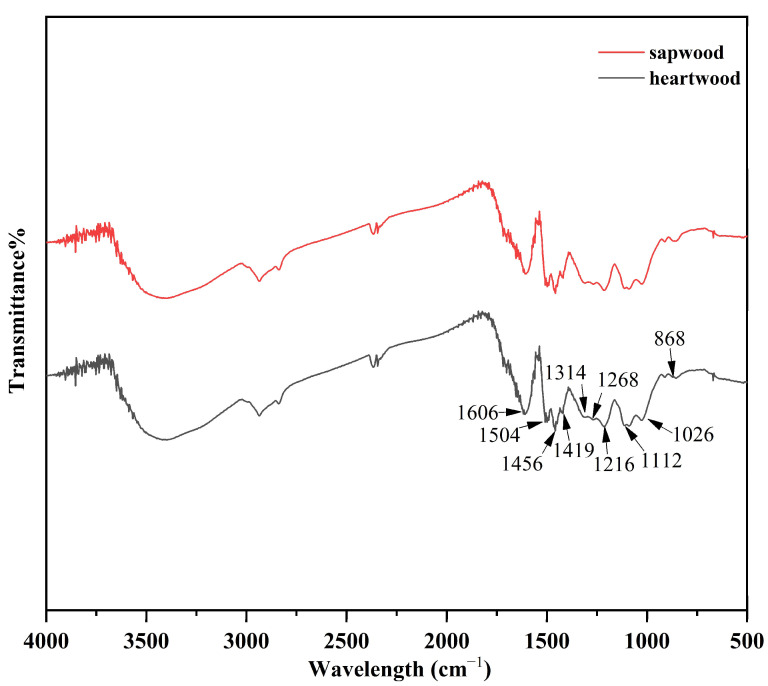
Infrared spectrum of Klason lignin of *D. oliveri.*

As shown in Figure 1, the peak positions of the infrared spectra of Klason lignin in heartwood and sapwood of *D. oliveri* were basically identical. It showed that the chemical functional groups of Klason lignin in heartwood and sapwood were basically the same. The absorption peaks at wavelengths 1606, 1504, 1456, and 1419 cm^−1^ belong to the vibrational absorption of the aromatic skeleton. The absorption peaks at 1268, 1026, and 868 cm^−1^ were assigned to guaiacyl units. The absorption peaks at 1314, 1216, and 1112 cm^−1^ were from the syringyl unit. This shows that the heartwood lignin of *D. oliveri* is mainly composed of guaiac-based units and syring-based units, which is the same type of lignin reported in *Dalbergia odorifera* [23].

The main factors affecting the color of wood are lignin and wood extracts [24]. The difference in lignin content and functional group structure between heartwood and sapwood was not obvious. Therefore, it is speculated that lignin may not be the main reason for the obvious color difference of *D. oliveri.* Previous studies had found that in several larch species, a* (reddish) and L* (brightness) parameters were highly correlated with wood extractive content, while b* (yellowish) parameters were closely related to cell wall chemical composition (cellulose, hemicellulose, and lignin) [25]. This indicated that the extract may be the main reason for the obvious color difference between heartwood and sapwood when the heartwood is red in color.

### 2.2. UV-Visible Spectroscopy of D. oliveri Extracts

**Figure 2:**
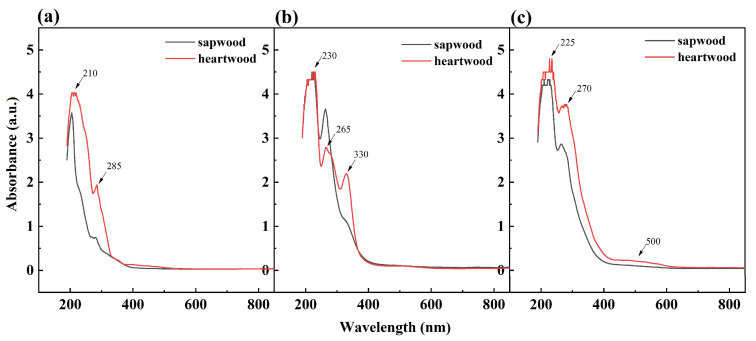
UV spectrum of *D. oliveri* heartwood and sapwood extract. (**a**) petroleum ether layer, (**b**) water layer, (**c**) ethyl acetate layer.

As shown in Figure 2, there were two peaks between 200–290 nm for the alcoholic extracts of heartwood and sapwood petroleum ether layer, and the peaks were near 210 and 285 nm, respectively. It indicated that the characteristic peaks of aromatic compounds exist in heartwood and sapwood of the petroleum ether layer extract, and the content of heartwood was higher than that of sapwood. Heartwood and sapwood water layer alcohol extracts had absorption peaks near 230, 265, and 330 nm in the ultraviolet region, but heartwood had a stronger absorption peak near 330 nm, while it was a shoulder peak in the sapwood. It shows that the chromophoric system between heartwood and sapwood in the water layer extract is roughly the same. It all contains chromophoric groups, such as benzene rings and conjugated double bonds, while the content of chromophoric groups in heartwood was higher. Heartwood and sapwood of the alcoholic extract of the ethyl acetate layer had absorption peaks near 225 and 270 nm. In addition, the heartwood had a weak and broad absorption peak at 400–600 nm, while sapwood had no absorption peak in the visible region. This indicates that chromogenic groups such as benzene rings exist in both heartwood and sapwood, while there was a long conjugate chain or polycyclic aromatic hydrocarbons at 400-600 nm in heartwood [26]. Therefore, heartwood had different chromogenic groups from sapwood.

The difference in wood color is associated with filled or deposited extracts in the cell wall [27]. Fen et al. [28] conducted an extensive targeted metabolic analysis of Taxus L heartwood and sapwood extracts and found significant differences in flavonoid substances, and 71 flavonoid compounds related to wood color were identified in heartwood using liquid chromatography-electrospray ionization-mass spectrometry (LC-EI-MS) technology. Flavonoids usually have obvious absorption peaks around 230–290 nm or 300–350 nm, and anthocyanins have two peaks between 200 and 700 nm, one at 500–600 nm in the visible light region, and the other is around 275 nm in the UV region. But most phenols have no absorption at 500 nm [29]. Thus, it is preliminarily determined that the main chromophoric substances in heartwood and sapwood of *D. oliveri* are flavonoid pigments. However, there are anthocyanins in heartwood, but not in sapwood, and the content of chromophoric groups in heartwood is also higher than that in sapwood. It may be the main reason for the obvious difference in color between heartwood and sapwood, and will be further identified by UHPLC-MS.

### 2.3. Identification of Pigment Components in D. oliveri Heartwood and Sapwood Extract

The chemical constituents of heartwood and sapwood for *D. oliveri* were explored by UHPLC-MS, and a total of 21 (details of MS/MS spectra and structural identification are given in Supplemental Figure) flavonoids were identified (Table 2). These include: seven flavonoids (orientin, vitexin, hispidulin, scrophulein, chrysin, 5-hydroxy-6,7-dimethoxy-2-phenyl-4H-chromen-4-one, diosmetin), eight isoflavones (daidzin, ononin, daidzein, glycitin, genistein, glycidein, formononetin, biochanin A), one flavonol (isorhamnetin), 3 flavanones (liquiritigenin, 2-(3,4-dihydroxyphenyl)-7-hydroxy-3,4-dihydro-2H-1-benzopyran-4-one, naringenin), one chalcone (isoliquiritigenin), and one anthocyanin (pelargonidin). The unique ingredients of sapwood of *D. oliveri* are daidzin, ononin, and glycitin. The unique components of heartwood are vitexin, isorhamnetin, and pelargonidin. 

#### 2.3.1. Flavonoids

**Figure 3:**
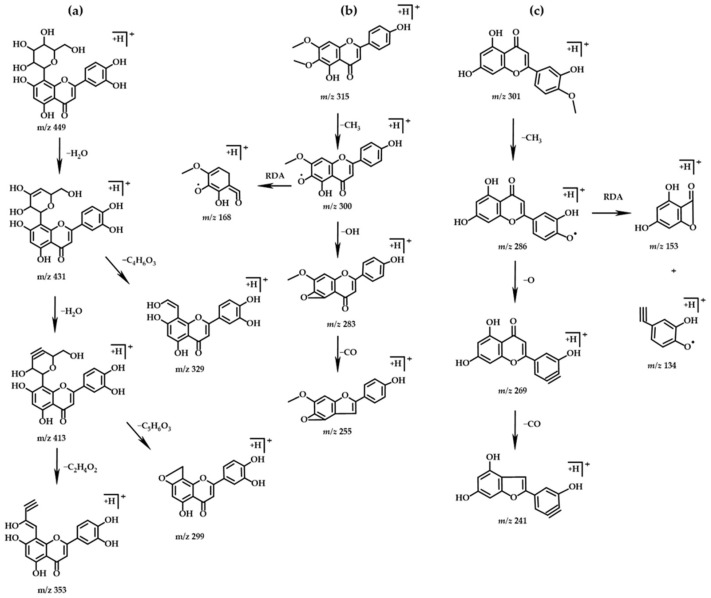
Chemical structure and fragmentation pathways of (**a**) orientin, (**b**) scrophulein, and (**c**) diosmetin.

Compounds 1 (tR = 4.68) and 2 (tR = 5.38) were identified as orientin and vitexin, both flavonoid monosaccharide carbon glycosides, which were relatively stable between the A, B, and C rings and undergo cleavage mainly through the breaking of their glycosidic bonds. Taking orientin as an example, its excimer ion peak was *m/z* 449 ([M+H]^+^), with a possible molecular formula of C_21_H_20_O_11_. The MS^2^ characteristic fragment ions at *m/z* 431, *m/z* 413, *m/z* 353, *m/z* 329, and *m/z* 299 are consistent with the data reported in the literature for this compound [30], and its possible cleavage pathway was shown in Figure 3.

Compounds 5 (tR = 7.46), 10 (tR = 9.29), 11 (tR = 9.47), 17 (tR = 10.51), and 18 (tR = 10.52) were identified as hispidulin, scrophulein, chrysin, 5-hydroxy-6,7-dimethoxy-2-phenyl-4H-chromen-4-one, and diosmetin, all of which were flavonoids, and the main cleavage mode was the easy loss of neutral small molecules such as H_2_O, CO_2_, CO, etc. The C ring is prone to generate characteristic fragment ions by the Retro Diels–Alder (RDA) cleavage. Taking scrophulein and diosmetin as examples, the scrophulein’s molecular formula was probably C_17_H_14_O_6_, and the excimer ion peak in positive ion mode was *m*/*z* 315 ([M+H]^+^), which upon fragmentation yields the product ions at *m/z* 300, *m/z* 283, *m/z* 255, and *m/z* 168, as reported in the literature [31], and its possible cleavage pathway was shown in Figure 3. Diosmetin had an excimer ion peak at *m/z* 301 ([M+H]^+^), and its molecular formula was probably C_16_H_12_O_6_. The MS^2^ characteristic fragment ions at *m/z* 286, *m/z* 269, *m/z* 241, *m/z* 153, and *m/z* 134, which are consistent with the data reported in the literature for this compound [32], and its possible cleavage pathway was shown in Figure 3.

#### 2.3.2. Isoflavones

**Figure 4:**
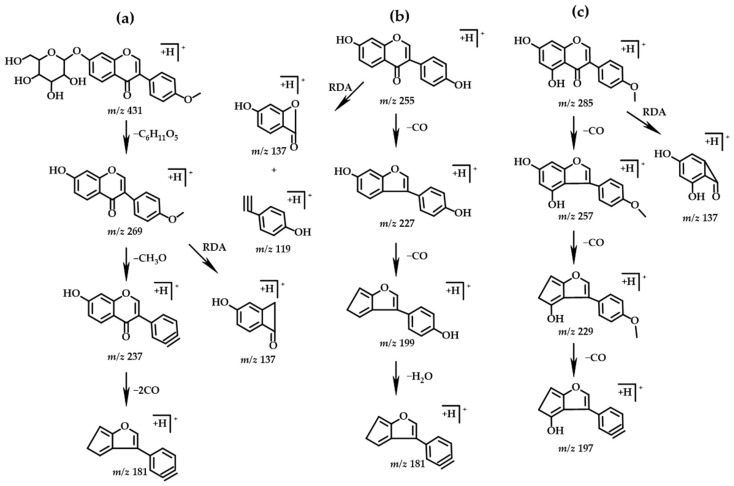
Chemical structure and fragmentation pathways of (**a**) ononin, (**b**) daidzin, and (**c**) biochanin A.

Compounds 3 (tR = 5.52), 7 (tR = 8.46), and 14 (tR = 9.96) were identified as daidzin, ononin, and glycitin, all of which were isoflavone monosaccharide glucoside compounds, and the main cleavage mode is the loss of one molecule of glucoside followed by the loss of neutral molecules such as H_2_O, CO_2_, and CO, etc. Taking ononin as an example, the excimer ion peak at *m/z* 431 ([M+H]^+^), which upon fragmentation yields the product ions at *m/z* 269, *m/z* 237, *m/z* 181, and *m/z* 137, which are inferred from the literature [33] and the database, and its possible cleavage pathway was shown in Figure 4.

Compounds 9 (tR = 9.04), 15 (tR = 10.12), 16 (tR = 10.41), 20 (tR = 11.52), 21 (tR = 12.78) were identified as daidzein, genistein, glycidein, formononetin, and biochanin A, all of which are isoflavones. The main cracking method was easily lost neutral molecules such as H_2_O, CO_2_, CO, etc. When there was a methoxy group substitution, it was easy to lose a molecule of CH_3_. The RDA cleavage of the C ring was mainly affected by the substitution positions on the A and B rings. Taking daidzein and biochanin A as examples, the excimer ion peak of daidzein in positive ion mode was *m*/*z* 255 ([M+H]^+^), the molecular formula was probably C_15_H_10_O_4_, and the MS^2^ characteristic fragment ions at *m*/*z* 227, *m*/*z* 199, *m*/*z* 181, *m*/*z* 137, and *m*/*z* 119. Combined with the retention time of the reference substance and the database, the compound was identified as daidzein, and its possible cleavage pathway is shown in Figure 4. In the positive ion mode, the excimer ion peak of biochanin A is *m*/*z* 285 ([M+H]^+^), the molecular formula is probably C_16_H_12_O_5_, and the MS^2^ characteristic fragment ions at *m*/*z* 257, *m*/*z* 229, *m*/*z* 197, and *m*/*z* 137, and its possible cleavage pathway is shown in Figure 4. Furthermore, the retention time of this compound matches well with that of biochanin A standard.

#### 2.3.3. Flavonol

**Figure 5:**
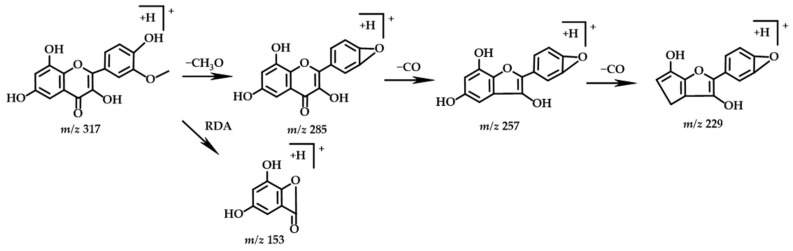
Chemical structure and fragmentation pathways of isorhamnetin.

Compound 6 (tR = 8.14) had an excimer ion peak of *m*/*z* 317 ([M+H]^+^), and its molecular formula is presumably C_16_H_12_O_7_. The MS^2^ fragment at the *m*/*z* values of 285, 257, 229, and 153 were inferred from the literature [34], and the database identified the compound as isorhamnetin, and its possible cleavage pathway is shown in Figure 5.

#### 2.3.4. Flavanones

**Figure 6:**
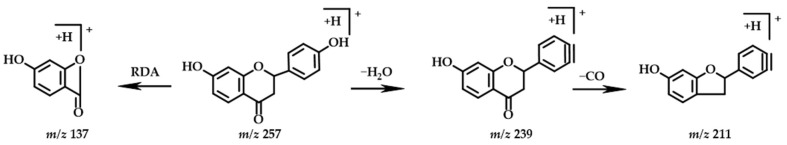
Chemical structure and fragmentation pathways of liquiritigenin.

Compounds 4 (tR = 7.34), 8 (tR = 8.51), and 13 (tR = 9.73) are 2-(3,4-dihydroxyphenyl)-7-hydroxy-3,4-dihydro-2H-1-benzopyran-4-one, liquiritigenin, and naringenin, all of which are dihydroflavonoids. The main cleavage method is RDA cleavage; the C ring 1 and 3 bonds are broken, and neutral molecules H_2_O, CO_2_, CO, etc. are also lost. Taking liquiritigenin as an example, the excimer ion peak in positive ion mode is *m/z* 257 ([M+H]^+^), the molecular formula was inferred to be C_15_H_12_O_4_, and the MS^2^ characteristic fragment ions at *m/z* 239, *m/z* 211, and *m/z* 137. The compound was identified as liquiritigenin based on the retention time of the reference substance and the database, and its possible cleavage pathway is shown in Figure 6.

#### 2.3.5. Chalcone

**Figure 7:**
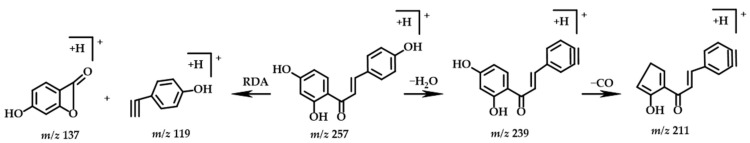
Chemical structure and fragmentation pathways of isoliquiritigenin.

Compound 19 (tR = 11.13) had an excimer ion peak of *m/z* 257 ([M+H]^+^), and its molecular formula is presumably C_15_H_12_O_4_. The MS^2^ characteristic fragment ions of *m/z* 239, *m/z* 211, *m/z* 137, and *m/z* 119, which are inferred from the retention time of the reference substance and the database. The compound was identified as isoliquiritigenin, and its possible cleavage pathway is shown in Figure 7.

#### 2.3.6. Anthocyanin

**Figure 8:**
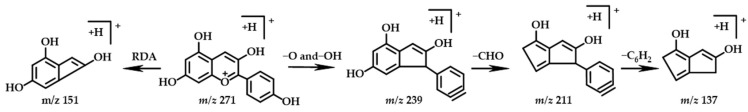
Chemical structure and fragmentation pathways of pelargonidin.

Compound 12 (tR = 9.61) had an excimer ion peak of *m/z* 271 ([M+H]^+^), and the molecular formula was presumed to be C_15_H_10_O_5_, with MS^2^ characteristic fragment ions at the *m/z* values of 239, 211, 151, and 137, which are inferred from the literature [35] and the database. The compound was identified as pelargonidin, and its possible cleavage pathway is shown in Figure 8.

### 2.4. Analysis of the Causes of Color Difference between D. oliveri Heartwood and Sapwood

#### 2.4.1. Different Pigment Components between Heartwood and Sapwood of *D. oliveri*

The unique pigment components of sapwood of *D. oliveri* were daidzin, ononin, and glycitin, all of which were isoflavone glycosides, and their color state was white, while the unique pigment component of heartwood was vitexin (flavonoid carbon glycosides) and isorhamnetin (flavonol). Its color state was yellow. Pelargonidin (anthocyanin) was red, and sapwood has no anthocyanin pigment, which is consistent with the results of UV spectroscopy (see Table 3).

This research confirmed that the main factor affecting plant color was the content and type of pigment molecules [36]. Wang et al. [37] reported that anthocyanins are the main coloring substances in the magnolia of purplish red and purple, while white and yellow-green magnolias contain no anthocyanins. In addition, anthocyanins have been reported to be the main pigments in red fruits [38,39]. Flavonoids and flavonols can form stable and complex pigment complexes with anthocyanins as copigments [40]. The color of pelargonidin was red, which was consistent with the color of *D. oliveri*. As flavonoid carbon glycosides and flavonols, vitexin and isorhamnetin have carbonyl auxochrome groups located at the 7 and 4 positions in their own structures, which can promote electron transfer and rearrangement, deepening the color of the compound. In addition, it can also combine with anthocyanins to form stable pigments [41]. In conclusion, pelargonidin, vitexin, and isorhamnetin can be used as potential markers for the color difference between heartwood and sapwood of *D. oliveri*.

#### 2.4.2. Differences in the content of main chemical components between heartwood and sapwood of *D. oliveri*

It can be seen from Table 4 that the content of heartwood pigment components liquiritigenin, genistein, isoliquiritigenin, formononetin, and biochanin A were 293.6505 ug/g, 161.8764 ug/g, 4787.8213 ug/g, 2133.5930 ug/g, and 2000.0880 ug/g. The content in sapwood was 31.7227 ug/g, 58.3365 ug/g, 18.9532 ug/g, 57.3800 ug/g, and 3.43030 ug/g, and the content of pigment in heartwood was 9, 3, 253, 37, and 583 times higher than that in sapwood, respectively. The results showed that the content of the pigment components isoliquiritigenin, formononetin, and biochanin A in heartwood was significantly higher than that in sapwood.

In another study conducted by Qiu et al., the content of compounds in heartwood and sapwood of teak (*Tectona grandis*) was significantly different, which was consistent with the results obtained in this paper that the content of chemical components in heartwood was greater than that in sapwood [42]. Isoliquiritigenin belongs to the chalcone, and its color state was yellow. It is an important substrate for the synthesis of other flavonoids and can form anthocyanins through a series of transformations by chalcone isomerase [43]. Formononetin and biochanin A are isoflavones, and their color state is yellow. There are carbonyl, methoxy, and other auxochromic groups related to wood color in the structure. Therefore, isoliquiritigenin, formononetin, and biochanin A may be the main pigment components to cause the color difference between heartwood and sapwood.

## 3. Materials and Methods

### 3.1. Materials and Preparation of Extracts

*Dalbergia oliver* shavings were taken from Laoyongchun Hongmu Furniture Factory, Nanning, China. It was identified by the Guangxi University Testing Centre as Dalbergia oliver heartwood and sapwood. They were then ground and passed through a 0.3 mm sieve, and these samples were used for the determination of the main chemical composition.

First, 1 g of dried wood powder was accurately weighed into a conical flask, and 30 mL 70% (*v*/*v*) of ethanol was added to it. Then, ultrasonic extraction was performed for 30 min; this process was repeated one more time. Subsequently, the extract was filtered and evaporated until dry by rotary evaporation under a vacuum to obtain the concentrated solution. The suspension solution was prepared by adding distilled water to the concentrate. After that, extraction was performed using petroleum ether and ethyl acetate, in that order. Three solutions were collected, concentrated, and evaporated to obtain the extract of the sample. These samples were used for extract analysis.

The dried sample (0.05 g) was precisely weighed and added to an 80% (*v*/*v*) methanol solution (5 mL), following which the extraction was performed by sonication for 40 min (40 kHz). The extraction solution (0.1 mL) was diluted 10 times with methanol and passed through a 0.22 µm microporous filter membrane, and then placed in a chromatography sample bottle. These samples were used to extract UHPLC-MS analysis.

### 3.2. Reagents and Standard

Chromatographically pure methanol, formic acid; standard products: formononetin, daidzein, isoliquiritigenin, liquiritigenin, genistein, chickpea A; all purity > 97%.

### 3.3. Determination of Chemical Composition

Determination of α-cellulose and hemicellulose content was measured by reference to the Chinese national standards according to GBT 2677.10-1995, “Fibrous raw material-Determination of Holocellulose” and GBT 744-1989, “Pulps-Determination of α-cellulose”, respectively.

The determination of lignin was carried out in accordance with the National Renewable Energy Laboratory (NREL) NREL/TP-510-42618, Determination of structural carbohydrates and lignin in biomass.

### 3.4. Fourier Infrared Spectroscopy (FTIR) Analysis of Lignin

The spectral characteristics of the lignin were determined using Fourier infrared spectroscopy (Thermo Fisher Scientific, Waltham, MA, USA). The sample was carefully ground, and 1–2 mg of the sample was ground with 0.1 g potassium bromide. The mixture was crushed into a preform. The analysis was performed in the measurement range of 400–4000 cm^−1^ with 32 cumulative scans.

### 3.5. UV Spectroscopic Analysis

The extract was measured using a UV spectrophotometer (Shimadzu Corporation, Shanghai, China). The analysis was ensured in the measurement range of 190–1100 cm^−1^. The resolution and sampling time of the instrument was set to 3.3 nm and 1 ms, respectively. Spectral data for the sample was collected and analyzed.

### 3.6. Ultra-High Performance Liquid Chromatography-Mass Spectrometry (UHPLC-MS) Analysis

The chemical composition of the samples was identified by UHPLC-MS (Thermo Fisher Scientific, Waltham, MA, USA). The chromatographic column was ACQUITY UPLC BEH C18 (2.1 mm × 50 mm × 1.7 μm). The ratio of formic acid and water was 1:1000 in mobile phase A. Methanol was chosen as mobile phase B. Gradient elution conditions were as follows: 0–15.0 min, 5% B; 15.0–18.0 min, 5–80% B; 18.0–18.1 min,100–5% B; 18.1–21 min, 5% B. The flow rate was 0.3 mL·min^−1^. The column temperature was 30 °C. The injection volume was 1 μL. The electrospray ionization was 300 °C. The transfer capillary temperature was 320 °C. The spray voltage in positive ion mode was 3.0 kV. The scan modes were selected as Full MS and Full MS/dd-MS^2^, respectively. The primary and secondary scan resolutions were 70,000 and 17,500. The flow rates of sheath gas and auxiliary gas were 206 and 69 kPa. The mass range was 100–1000 Da.

The content of the characteristic flavonoid component was determined by the external standard method. The standard working curve of flavonoids was established by the ratio of peak area and sample concentration.

### 3.7. Data Analysis

A *t*-test for the parameters of the chemical composition of the wood was run in SPSS package ver.24.0.0.0. The identification of chemical components uses the XIC Manager module in the Compound Discoverer 3.1 software (Thermo Fisher Scientific, Waltham, MA, USA). for automatic screening and analysis of accurate relative molecular masses, combined with relevant literature, mzCloud Mass Spectral Library database, compound secondary mass spectrometry fragmentation rules, and retention times of chemical standards to infer their possible chemical structures.

## 4. Conclusions

The content of the main components in the heartwood and sapwood of *D. oliveri* was not obvious, and heartwood and sapwood were weakly acidic. The lignin content was similar between heartwood and sapwood, and the functional group structure was basically identical. The main color-emitting components of heartwood of *D. oliveri* are flavonoids and anthocyanins, and for sapwood they are flavonoids. The pigment components in heartwood and sapwood of *D. oliveri* were identified, and their main components were quantified. A total of 21 flavonoid compounds were identified from heartwood and sapwood, among which vitexin, isorhamnetin, pelargonidin, isoliquiritigenin, formononetin, and biochanin A may be the main pigment components affecting the color difference between heartwood and sapwood.

## Figures and Tables

**Table 1:** Main chemical components of sapwood and heartwood of *D. oliveri.*

	PH Value	Holocellulose (%)	α-Cellulose (%)	Klason Lignin (%)		
				Acid-Insoluble Lignin	Acid-Soluble Lignin	Total
sapwood	6.28	80.35 ± 1.0 ^a^	63.63 ± 0.3 ^a^	25.96 ± 0.6 ^a^	1.94 ± 0.1 ^a^	27.90 ± 0.6 ^a^
heartwood	5.92	71.81 ± 2.2 ^b^	66.81 ± 0.8 ^b^	26.79 ± 0.4 ^a^	1.45 ± 0.1 ^b^	28.24 ± 0.3 ^a^

Different letters mean significant differences at the 0.05 level.

**Table 2:** Pigment components of *D. oliveri* extract analyzed by UHPLC-MS.

No.	Structure Type	TR	Molecular Formula	Molecular Mass [M+H]^+^		MS^2^	Identification
				Theoretical	Measured		
1	Flavonoids	4.68	C_21_H_20_O_11_	449.1078	449.1091	431.0955, 413.0857, 353.0653, 329.0649, 299.0543	Orientin
2	Flavonoids	5.38	C_21_H_20_O_10_	433.1129	433.1124	415.1023, 397.0915, 313.0700, 283.0597, 255.0652	Vitexin
3	Isoflavones	5.52	C_21_H_20_O_9_	417.1180	417.1177	255.0634, 177.1118, 133.0858	Daidzin
4	Flavanones	7.34	C_15_H_12_O_5_	273.0758	273.0755	255.0649, 227.0703, 209.0584, 163.0387, 137.0232, 135.0438	2-(3,4-dihydroxyphenyl)-7-hydroxy-3,4-dihydro-2H-1-benzopyran-4-one
5	Flavonoids	7.46	C_16_H_12_O_6_	301.0707	301.0705	286.0469, 269.0439, 241.0491, 213.0544	Hispidulin
6	Flavonols	8.14	C_16_H_12_O_7_	317.0656	317.0654	285.0390, 257.0437, 229.0490, 153.0181	Isorhamnetin
7	Isoflavones	8.46	C_22_H_22_O_9_	431.1337	431.1334	269.0802, 237.0541, 237.0541 181.0645, 137.0231	Ononin
8 *	Flavanones	8.51	C_15_H_12_O_4_	257.0808	257.0807	239.0697, 211.0750, 137.0232	Liquiritigenin
9 *	Isoflavones	9.04	C_15_H_10_O_4_	255.0652	255.0651	227.0700, 199.0750, 181.0644, 137.0231, 119.0490	Daidzein
10	Flavonoids	9.29	C_17_H_14_O_6_	315.0863	315.0860	300.0621, 283.0595, 255.0645, 168.0567	Scrophulein
11	Flavonoids	9.47	C_15_H_10_O_4_	255.0652	255.0649	137.0231, 119.0492	Chrysin
12	Anthocyanin	9.61	C_15_H_10_O_5_	271.0601	271.0599	239.0700, 211.0755, 151.0389, 137.0232	Pelargonidin
13	Flavanones	9.73	C_15_H_12_O_5_	273.0758	273.0756	255.0655, 153.0181, 119.0493	Naringenin
14	Isoflavones	9.96	C_22_H_22_O_10_	447.1286	447.1285	285.0751, 241.0488, 213.0543	Glycitin
15 *	Isoflavones	10.12	C_15_H_10_O_5_	271.0601	271.0600	225.0542, 197.0590, 169.0645, 137.0232	Genistein
16	Isoflavones	10.41	C_16_H_12_O_5_	285.0758	285.0758	253.0488, 225.0542, 197.0595, 137.0231	Glycitein
17	Flavonoids	10.51	C_17_H_14_O_5_	299.0914	299.0916	285.0711, 283.0599, 167.0339, 119.0490	5-hydroxy-6,7-dimethoxy-2-phenyl-4H-chromen-4-one
18	Flavonoids	10.52	C_16_H_12_O_6_	301.0707	301.0707	286.0466, 269.0438, 241.0488, 153.0179, 134.0360	Diosmetin
19	Chalcone	11.13	C_15_H_12_O_4_	257.0808	257.0807	239.0699, 211.0752, 137.0232, 119.0492	Isoliquiritigenin
20 *	Isoflavones	11.52	C_16_H_12_O_4_	269.0808	269.0806	254.0565, 237.0544, 137.0234, 118.0414	Formononetin
21 *	Isoflavones	12.78	C_16_H_12_O_5_	285.0758	285.0757	257.0803, 229.0856, 197.0595, 137.0231	Biochanin A

Note: * Confirmed by comparison with the reference substance.

**Table 3:** Different chemical constituents between *D. oliveri* heartwood and sapwood.

	Structure	Compound	Color
sapwood	isoflavone	daidzin	white
	isoflavone	ononin	white
	isoflavone	glycitin	white
heartwood	flavonoid	vitexin	yellow
	flavonol	isorhamnetin	yellow
	anthocyanin	pelargonidin	red

**Table 4:** Content of main chemical components between heartwood and sapwood of *D. oliveri.*

Compound	Calibration Caves	R^2^	Heartwood (ug/g)	Sapwood (ug/g)
Liquiritigenin	y = 1,779,112.4834x + 16,661,916.1497	0.996	293.6505	31.7227
Genistein	y = 1,371,988.0761x + 14,319,545.4588	0.995	161.8764	58.3365
Isoliquiritigenin	y = 1,779,112.4834x + 16,661,916.1497	0.999	4787.8213	18.9532
Formononetin	y = 3,740,086.0193x + 16,632,919.1163	0.999	2133.5930	57.3800
Biochanin A	y = 2,293,814.2766x + 19,031,906.6542	0.997	2000.0880	3.4303

## Data Availability

Data are available from the corresponding author on request.

## Supplementary Materials

The following supporting information can be downloaded at: https://www.mdpi.com/article/10.3390/molecules27227978/s1, Supplemental Figure: Structural identification of flavonoid components in *D. oliveri*.
