# Supplementary figures and images for: Differences in Chemical Constituents between Dalbergia oliveri Heartwood and Sapwood and Their Effect on Wood Color
Source: Molecules. 2022 Nov 17;27(22):7978. doi: 10.3390/molecules27227978 (PMC9695600; doi:10.3390/molecules27227978)

Supplemental Figure: Structural identification of flavonoid components in *D. oliveri*

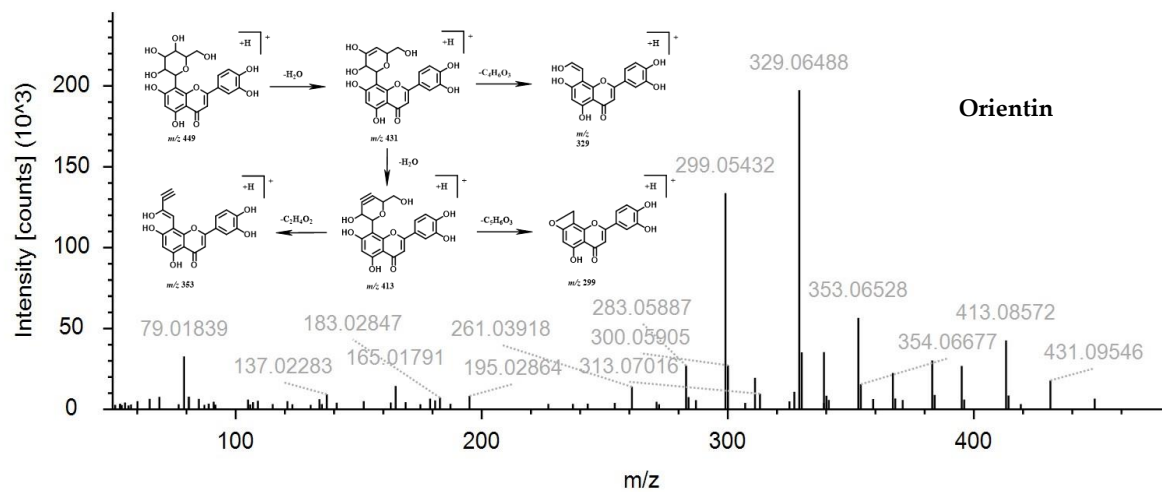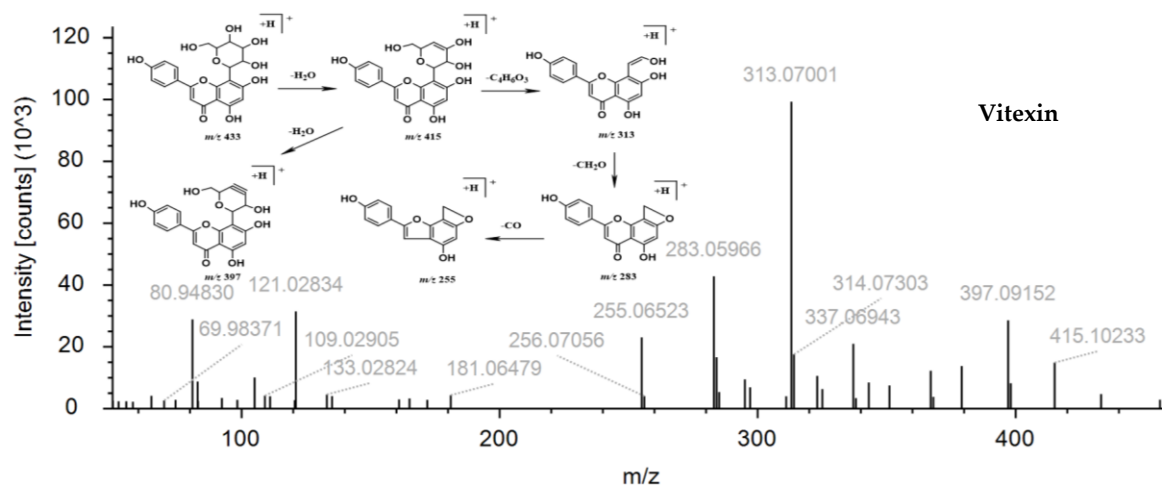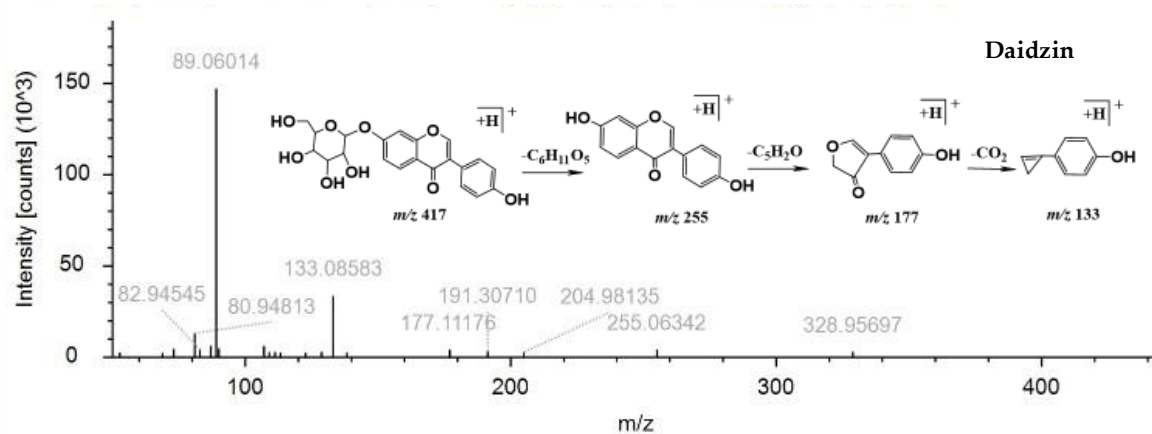

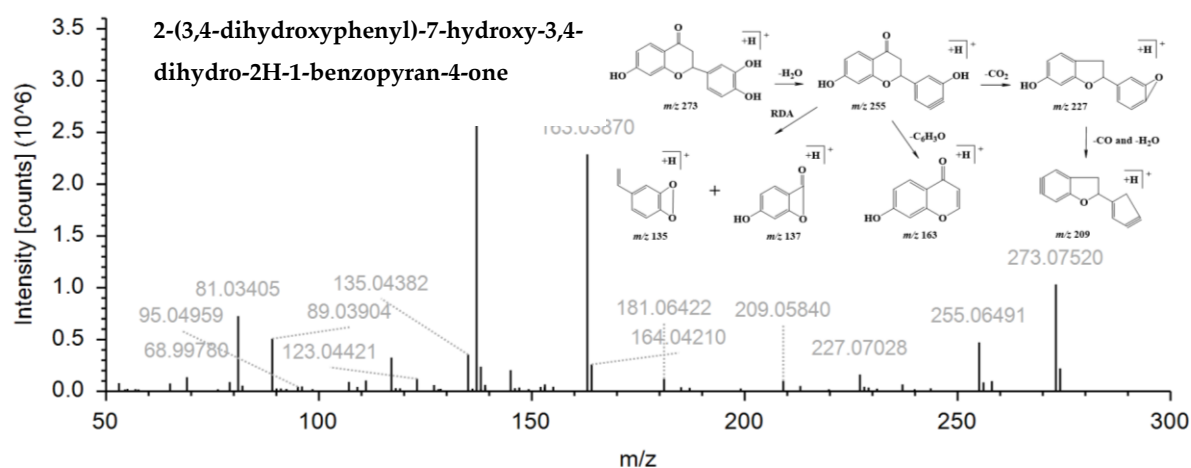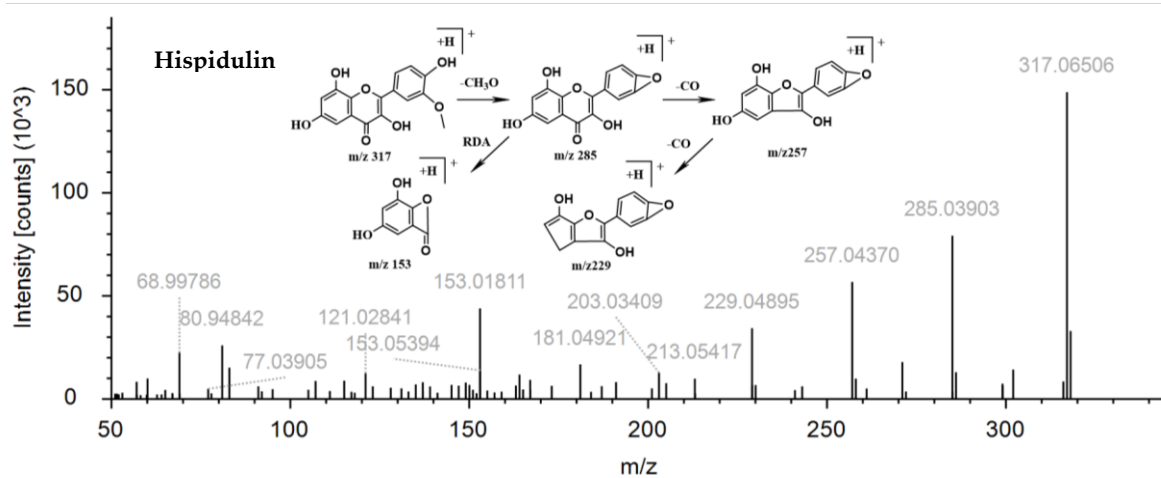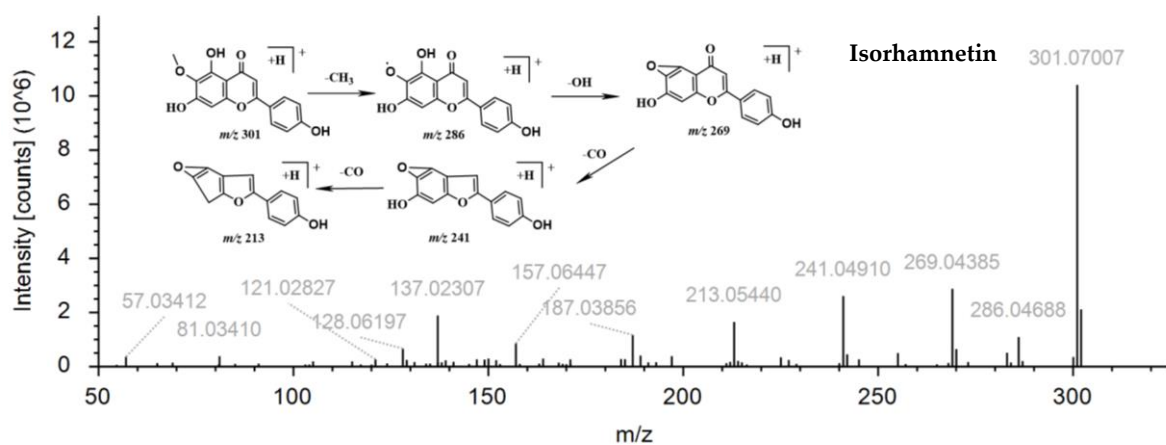

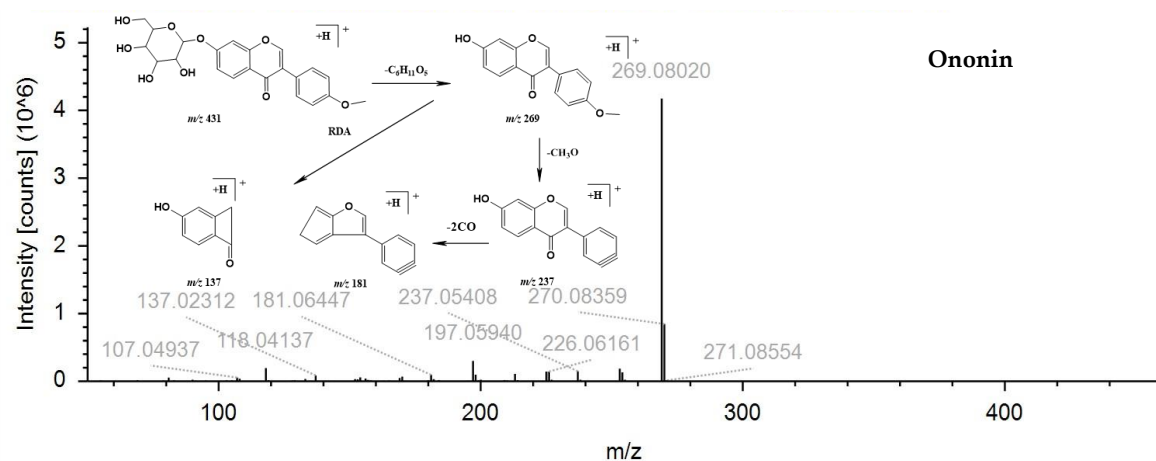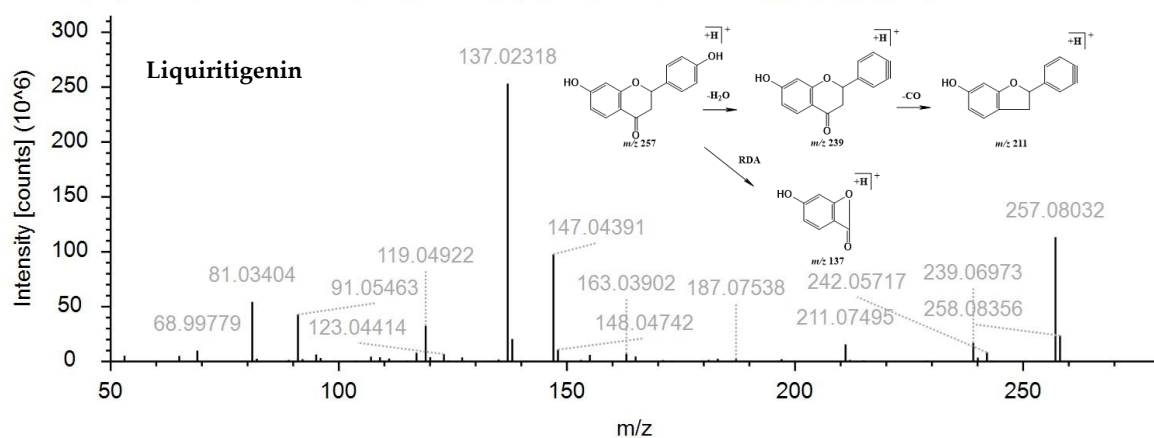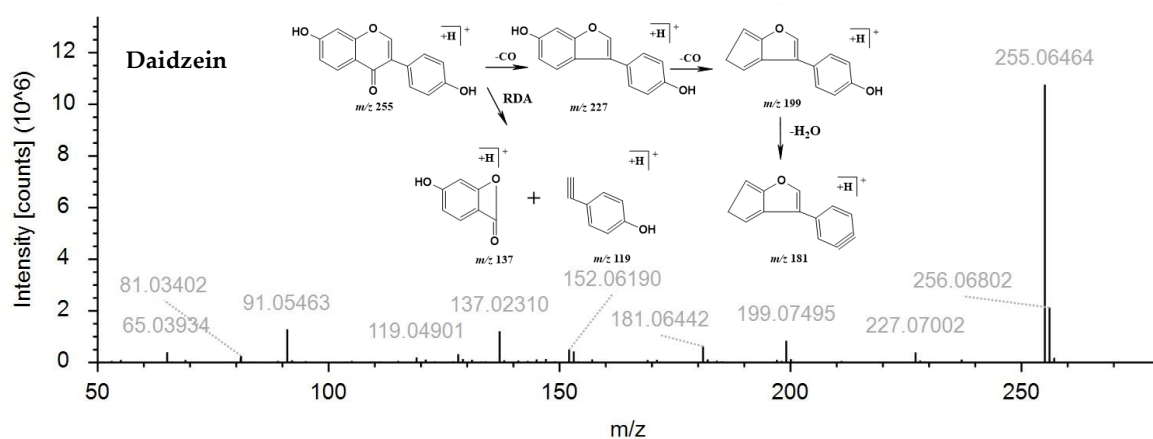

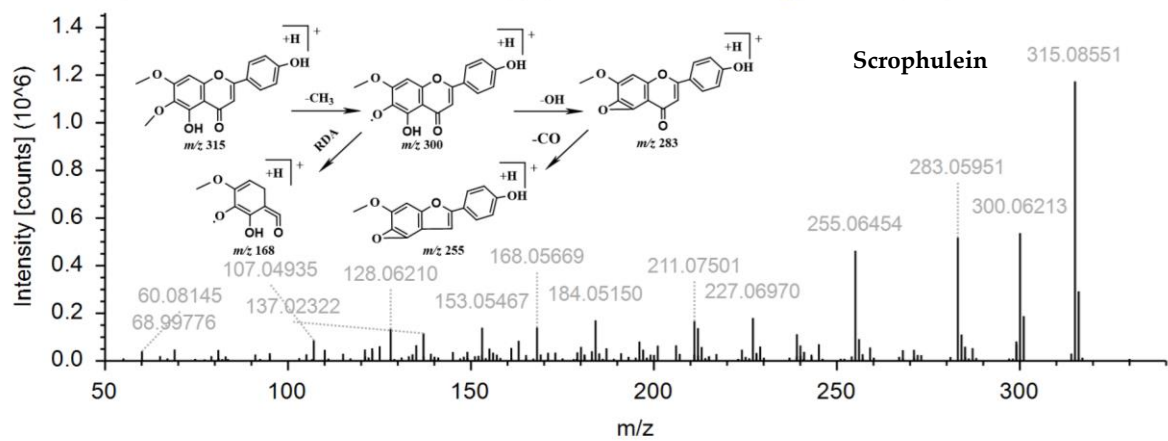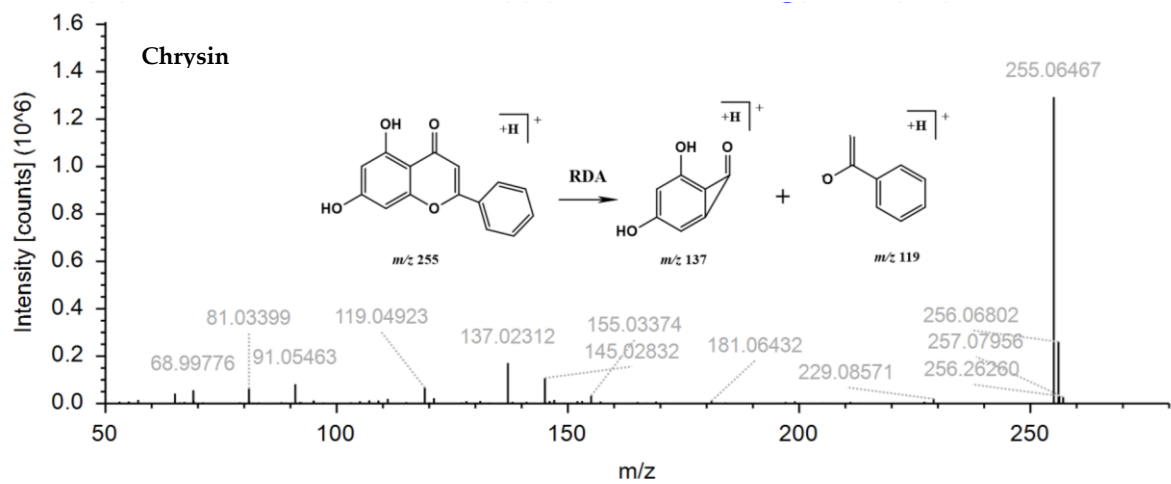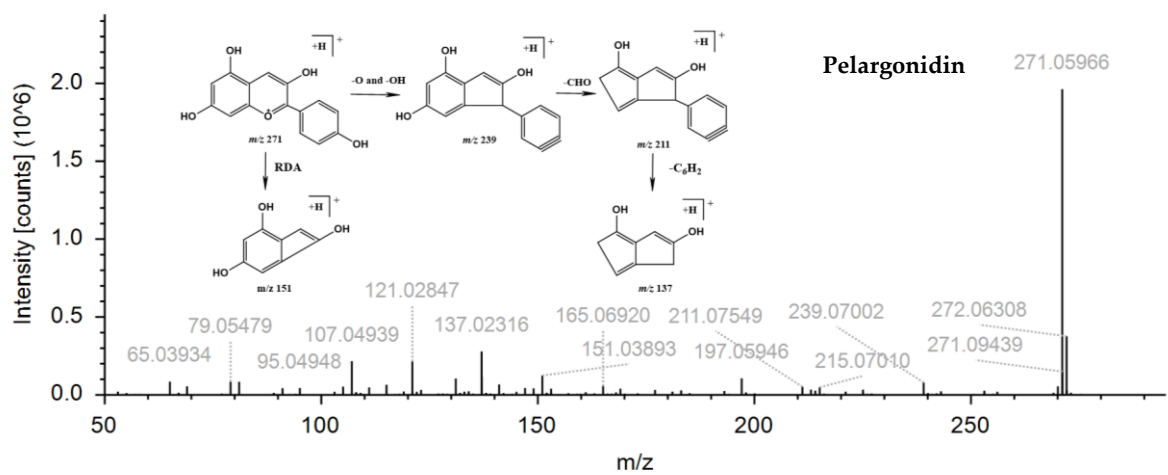

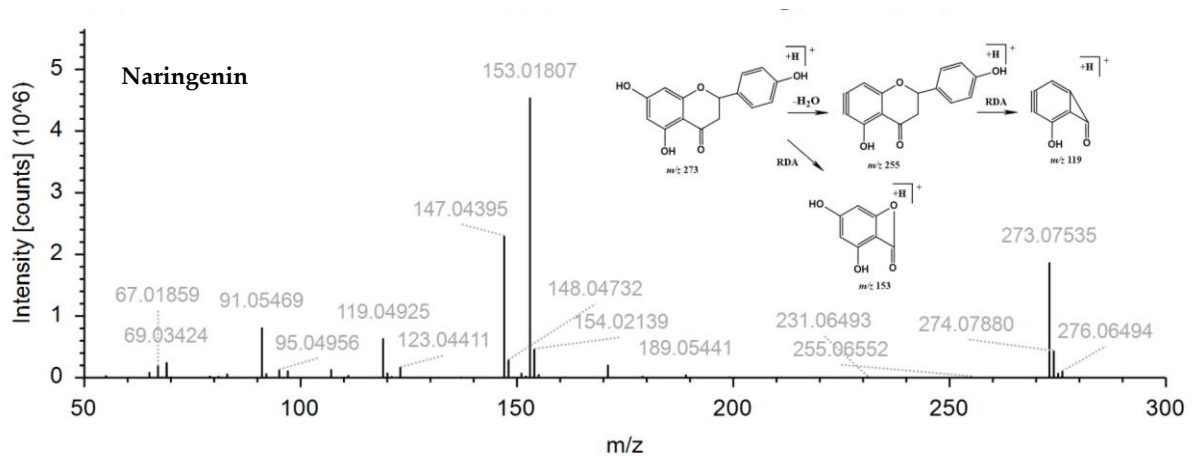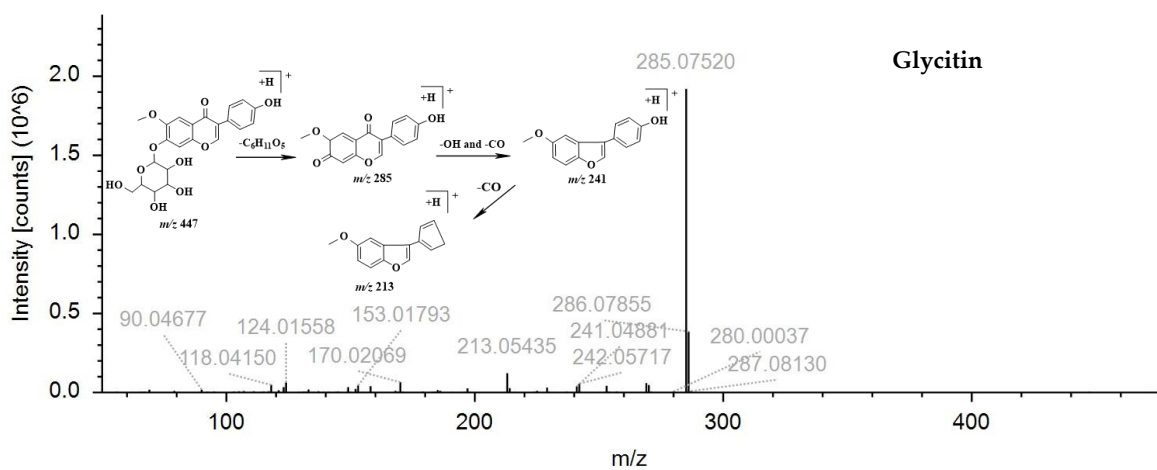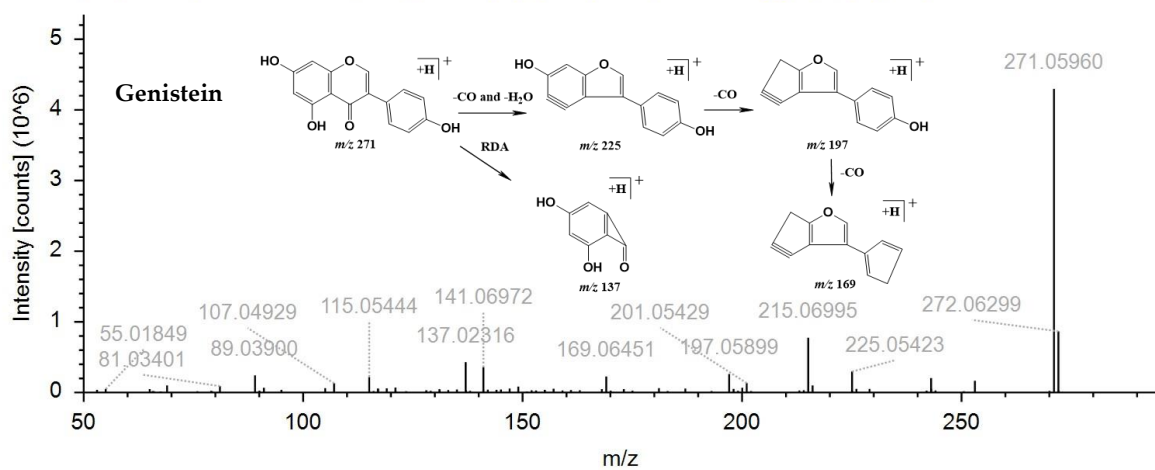

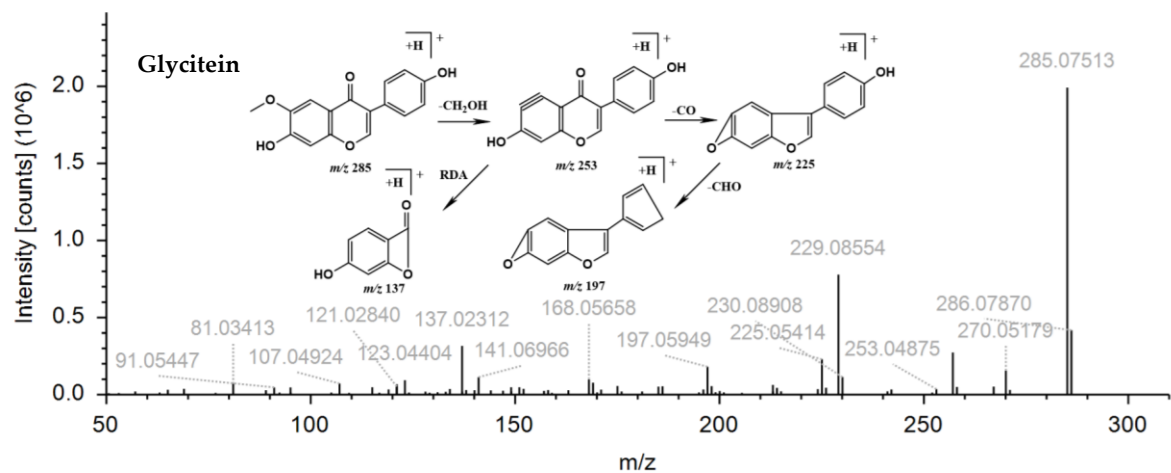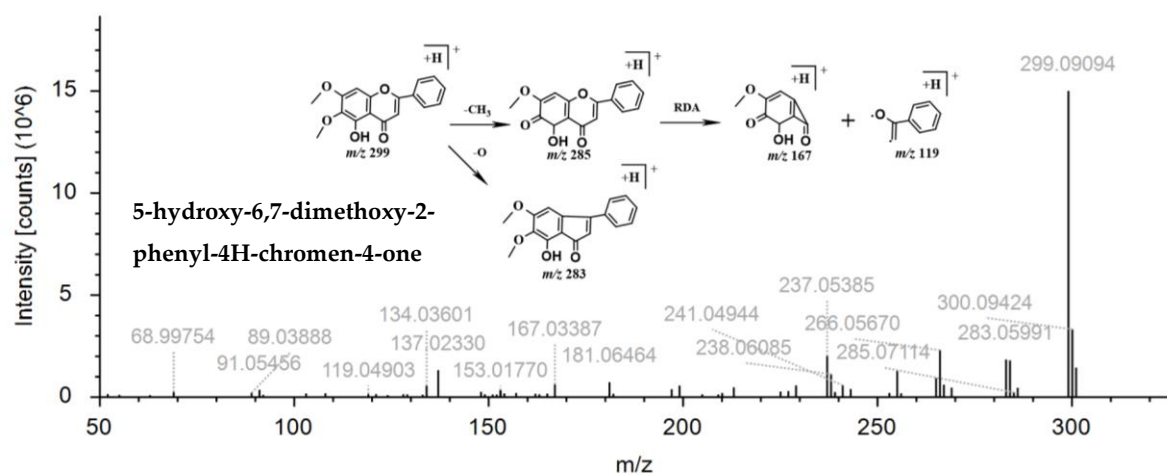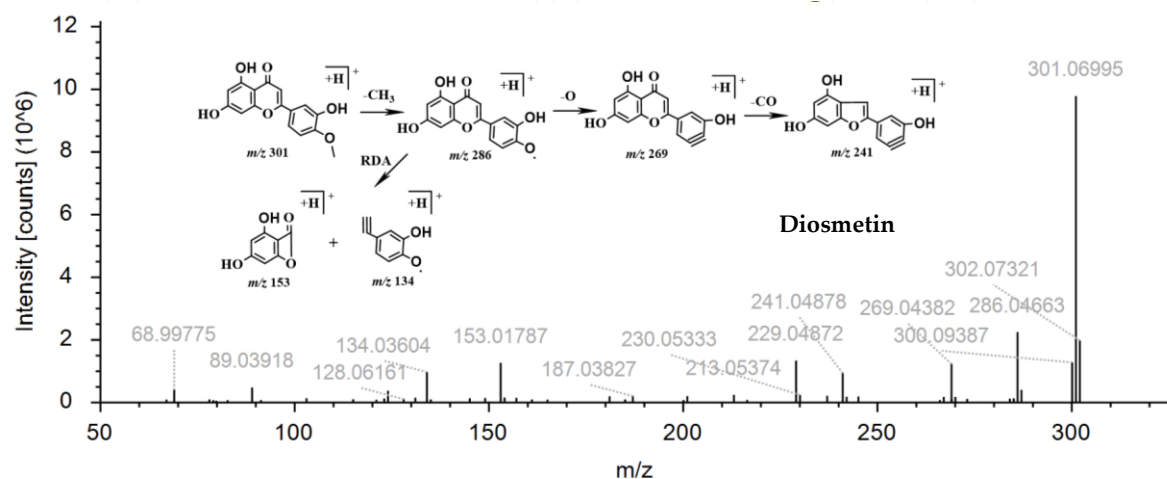

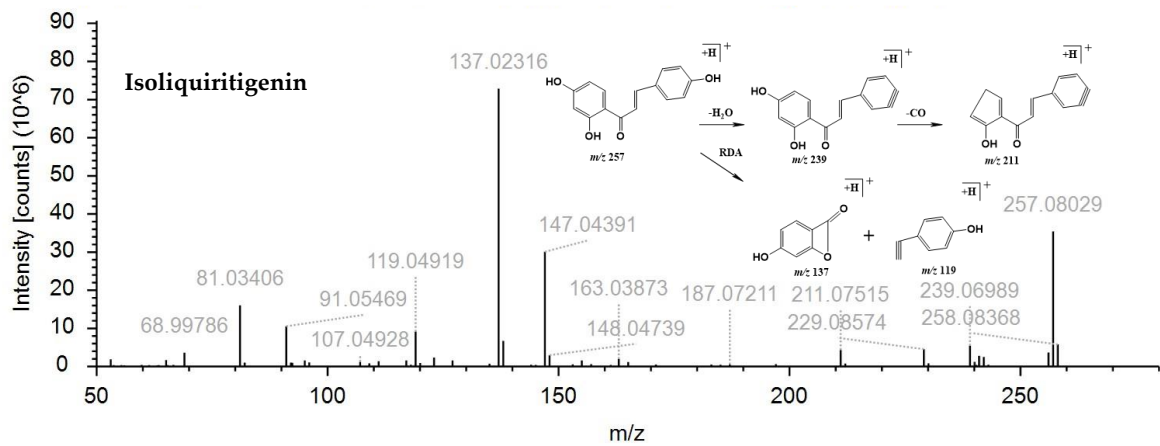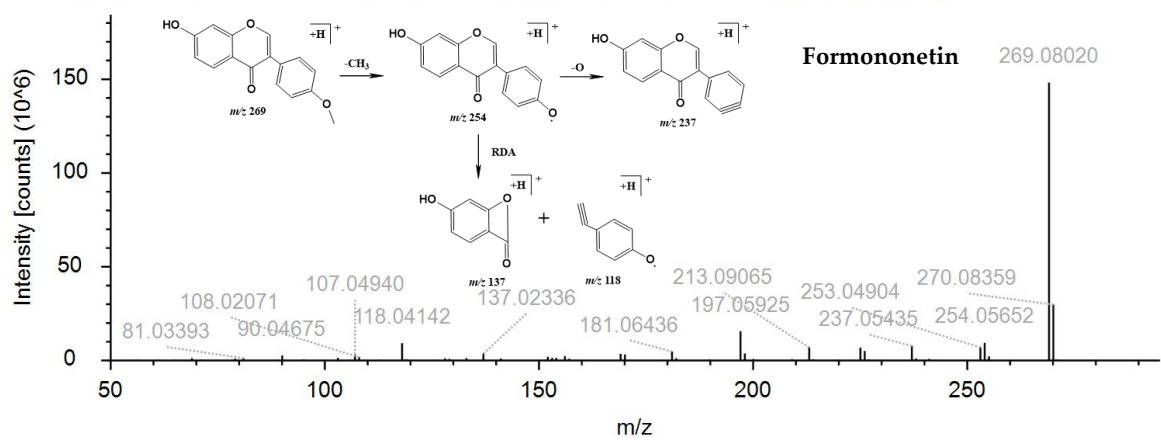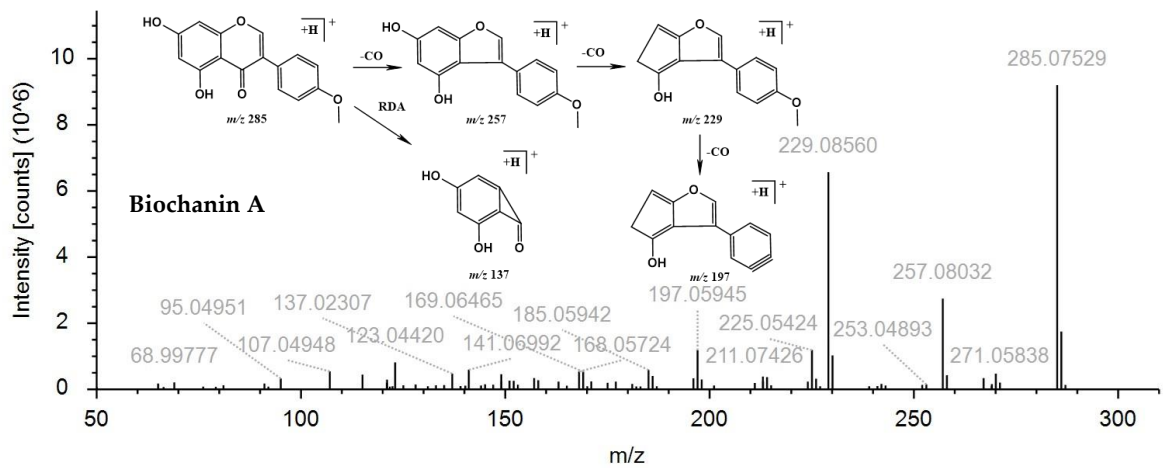

Supplement: Supplementary file 1 [file molecules-27-07978-s001.zip › molecules-1998971-supplementary.pdf]
